# Outcomes of Early Ligation of Patent Ductus Arteriosus in Preterms, Multicenter Experience

**DOI:** 10.1097/MD.0000000000000915

**Published:** 2015-06-26

**Authors:** Mohamed H. Ibrahim, Ahmed A. Azab, Naglaa M. Kamal, Mostafa A. Salama, Hatem H. Elshorbagy, Enas A.A. Abdallah, Abdulrahman Hammad, Laila M. Sherief

**Affiliations:** From the Benha Faculty of Medicine, Benha University (MHI, AAA, MAS); Faculty of Medicine, Cairo University, Cairo (NMK, EAAA); Menufia Faculty of Medicine, Menufia University (HHE); Al Azhar Faculty of Medicine, Al Azhar University (AH); Faculty of Medicine, Taif University (AAA); Faculty of Medicine, Zagazig University, Egypt (LMS); and Pediatric Department, Armed Forces Hospital-Taif, KSA (AAA, NMK, MAS, HHE).

## Abstract

Persistent ductal patency may have serious effects in preterm infants. Analysis of the results of different trials were inconclusive in determining whether medical or surgical closure of the ductus is preferable and what is the best timing for surgical intervention.

The aim of this study was to evaluate the effect of timing of surgical closure of patent ductus arteriosus (PDA) on ventilatory, hemodynamic, and nutritional status of preterm infants.

The authors retrospectively looked at the outcomes of surgical ligation of PDA from January 2010 to June 2014 at 2 Saudi neonatal intensive units at 2 tertiary care centers and the authors compared the results of early ligation (before 3 weeks) to the late ligation (after 3 weeks) regarding different hemodynamic, ventilatory, and nutritional parameters.

A total of 120 preemies were included (75 preemies with early ligation and 45 with late ligation of PDA). The early ligation group had shorter duration of assisted ventilation of 10 (8–37) days as compared with 37 (26–90) days in the late ligation group (*P* < 0.05). The median fraction of inspired oxygen, needed to maintain good oxygen saturation in patients, was higher in the late ligation group [0.29 (0.21–0.70)] than in the early group [0.23 (0.21–0.55)] at 24 hours postoperatively. Full oral feeding was achieved earlier in the early ligation group than in the late group, 29 (15–73) days of life versus 53 (34–118) days of life, respectively (*P* < 0.05). Body weight at 36 weeks postconceptional age was higher in the early group—2100 (1350–2800) g—than in the late group—1790 (1270–2300) g—(*P* < 0.05).

Our study demonstrated that earlier surgical ligation of the PDA in preterm infants has a more favorable nutritional and ventilatory outcome.

## INTRODUCTION

During the last 4 decades, there has been a marked increase in premature delivery in many countries. Infants with extremely low birth weight, 500 to 1500 g, represent approximately 1% of all live births and >60% of all neonatal deaths.^1^ The art and science of caring for extremely premature infant have developed to the point where survival of infants weighing no more than 500 g is not uncommon.^2^ Preterm infants with moderate to large left-to-right shunts through the patent ductus arteriosus (PDA) have a greater mortality rate than those without a PDA.^3^ They also have an increased risk of pulmonary edema and hemorrhage, bronchopulmonary dysplasia (BPD), and a decrease in perfusion and oxygen delivery to end-organs.^3^ The type and duration of the ventilatory support, drug therapy, phototherapy, blood transfusions, and diuretics undoubtedly account for the wide variations in the incidence reported from different institutions.^4^

The current management of PDA in premature infants includes 3 different approaches: conservative management with supportive therapy alone, pharmacologic closure using cyclooxygenase inhibitors (eg, indomethacin), and surgical ligation.^5^

There are no randomized controlled trials comparing outcomes of the 3 different approaches. Therefore, it remains unclear which approach is most advantageous for premature infants and whether clinical parameters or settings may favor one approach over another. This uncertainty has lead to variation in the management of PDA in preterm infants not only among different neonatal intensive care units (NICU), but often within a single NICU.^6^

Timing of shift to surgical ligation or surgical ligation from the start is an area of eager debate. The rationale of this work was to find out the effect of timing of surgical closure on cardiorespiratory and nutritional outcome in preterm babies. Postoperative complications, hospital stay, and mortality were included as secondary end point.

## PATIENTS AND METHODS

We carried a retrospective study at NICU departments of King Abdulaziz University Hospital and Alnoor Specialty Hospital, Saudi Arabia. It included all preterm infants (<32 weeks gestation) who required surgical closure of PDA between January 2010 and June 2014. The study was approved by the research and ethical committees of the contributing hospitals.

Patients with intrauterine growth retardation and major congenital malformations were excluded.

Analysis of Echo-Doppler studies was done, including two-dimensional, M-mode, and color Doppler echocardiographic images that were performed for every patient. The studies were done on GE vivid 7 and vivid 9 machines. The following parameters were obtained:

- Assessment of PDA size and direction of flow: from the parasternal short-axis and high left parasternal views using two-dimensional images and color flow mapping to get the narrowest diameter.^7^
- Assessment of the pulmonary arteries: measurements for the main, left, and right pulmonary artery diameters (mm) were obtained from parasternal short axis view and high left parasternal views. Pulsed Doppler images of the proximal left pulmonary artery were reviewed and its maximum Doppler velocity was taken.^7^
- Cardiac chambers measurement: left ventricular end-systolic and end-diastolic dimensions, left atrial, as well as aortic root dimension were obtained from the parasternal short-axis or long-axis view. Left ventricular fractional shortening was calculated.^7^
- Detection of other congenital anomalies that might be considered as exclusion criteria: criteria to accept surgical closure were failed medical therapy or when indomethacin therapy was contraindicated or hemodynamic instability during ongoing but incomplete medical treatment. The PDA must be hemodynamically significant judged either clinically by (mean arterial pressure less than gestational age in weeks, ventilator dependence, or heart failure symptoms); and/or by echocardiographic data: left atrial/aortic root ratio >1.6, the mean velocity in the left pulmonary artery >0.6m/s, and the PDA diameter >3 mm. All infants had an exclusively left to right shunting through the PDA.

Contraindications for indomethacin treatment included serum creatinine >1.7 mg/dL, active bleeding, sepsis, necrotizing enterocolitis (NEC), or oliguria. Contraindications for PDA ligation included severe pulmonary hypertension with predominant right-to-left or bidirectional shunt, life-threatening infection, and septic shock.

Timing of surgery was dependant on the availability of operative time (ie, the time schedule of the surgeons and anesthesiologists and on the availability of the operating room).

The chest was entered through a muscle-sparing left posterolateral minithoracotomy. The third intercostal space was entered, and the PDA was either ligated with transpleural double silk 2-ligature or clipped using single (usually medium sized) hemoclip. A postoperative chest tube was not a routine practice.

After collecting basic and clinical data, infants were divided into 2 groups for further analysis: early ligation group (surgery was performed before 3 weeks of life) and late ligation group (surgery was performed after 3 weeks).

Perioperative data included gestational age and weight, weight at time of surgery, delay time from birth to surgery, mean arterial pressure, requirement of inotropic support, and echocardiographic data.

Postoperative data that represent the short-term outcomes that are the mainstay aim of this study included ventilatory (respiratory) parameters, hemodynamic data, and nutritional status as primary outcomes and postoperative complications, mortality, and hospital stay as secondary outcomes.

Respiratory data collected were ventilator mode and fraction of inspired oxygen (FiO2) at 6, 12, 24, 48 hours, 7, and 28 days, then at 36 weeks of postconceptional age. BPD was defined as any requirement for supplemental oxygen at 36 weeks of conceptional age.

Hemodynamic data collected were focusing on the improvement of the mean arterial pressure on the same day of surgery (after 6 hours) and on the second day, 24 hours after surgery, also the need for inotropic support to maintain adequate perfusion and blood pressure after surgery was determined.

Nutritional status was assessed via age at oral feeding and body weight at 36 weeks of postconceptional age. Postoperative complications such as bleeding, persistent pneumothorax (2–5 days), superficial wound infection, and recurrent laryngeal nerve injury (hoarseness, stridor, difficult continuous positive airway pressure weaning) were noted.

## STATISTICAL ANALYSIS

The data were analyzed using statistical package of social science (SPSS, IBM, Chicago, IL, 6066–6307, USA) version 15 for Microsoft Windows.

Kolmogorov–Smirnov test was used to verify and check the normal distribution of parametric data. Chi-square or Fisher exact test was used for difference of proportions, and the Mann–Whitney *U* test to compare the 2 groups. Chi-square used to compare nonparametric data and independent *t*-test in parametric data between both groups. Results are expressed as median and range. Statistically significant difference occurred when *P* ≤ 0.05.

## RESULTS

Among 120 preterm infants with <32 weeks’ gestation included in our study, who underwent surgical closure of the PDA, 75 patients underwent surgical closure in the first 3 weeks of life and were placed in the first group, which is the early ligation group; the remaining 45 patients comprised the late ligation group.

The patients‘ characteristics, hemodynamic data, ventilatory requirements, and echocardiographic parameters of both groups were nearly similar (Tables 1–4). However, **t**he requirement for preoperative inotropes (eg, dobutamine and/or dopamine) was significantly higher in the late ligation group (62.3%) in comparison with (37.8%) in the early ligation group (*P* < 0.001), indicating that the patients with prolonged patency of PDA had a more risk of hemodynamic instability which was controlled by surgical ligation (Table 3).

**TABLE 1:**
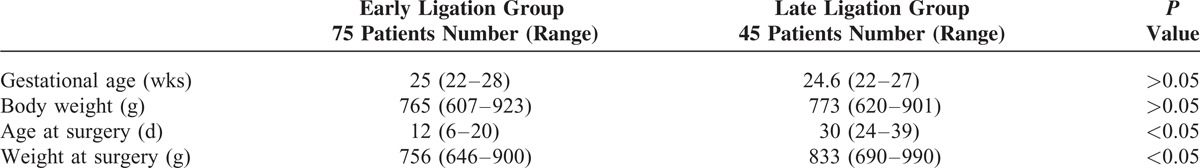
Demographic Data of the Study Patients

**TABLE 2:**
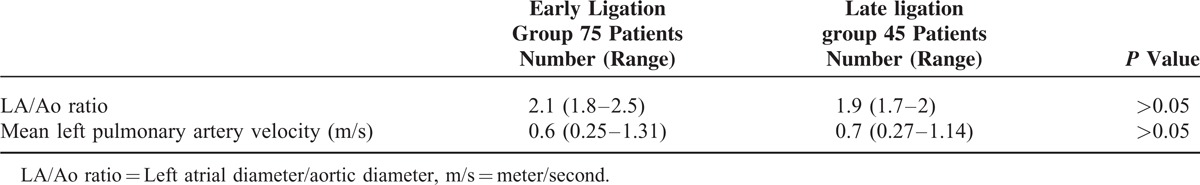
Echo Doppler Data

**TABLE 3:**
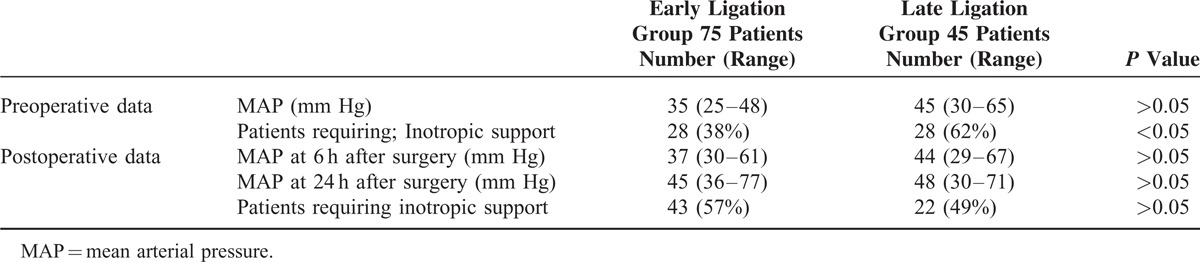
Hemodynamic Data

**TABLE 4:**
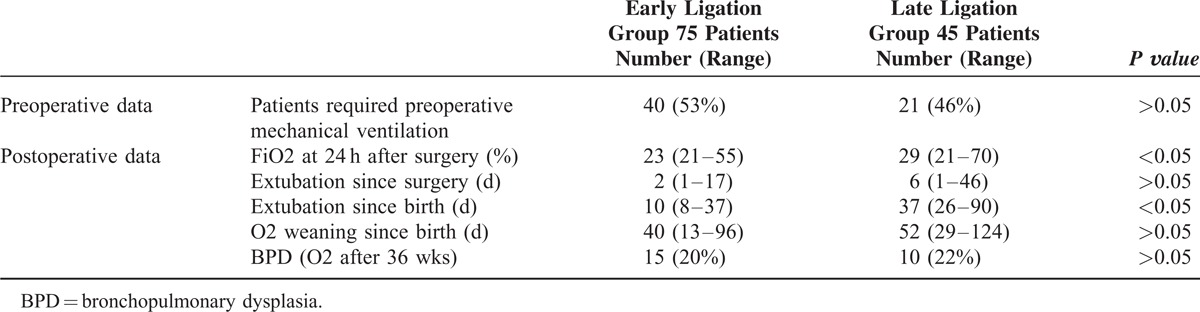
Respiratory Data

Hemodynamic data were similar in both groups after the surgery. In contrary to preoperative period, there was no significant difference in inotropic support in both groups after surgery (Table 3).

The total duration of assisted ventilation was significantly shorter in patients of the early ligation group with 10 (8–37) days than in patients of the late ligation group with 37 (26–90) days (*P* < 0.05) (Table 4).

The median FiO2 was higher in the late ligation group [0.29 (0.21–0.70)] than in the early group [0.23 (0.21–0.55)] at 24 hours postoperatively; however, the incidence of BPD did not differ between both groups (Table 4).

Nutritional status showed the most striking difference between both groups after surgery. Full oral feeding was acquired earlier in the early ligation group than in the late group, 29 (15–73) days of life versus 53 (34–118) days of life, respectively (*P* < 0.05). Body weight at 36 weeks postconceptional age was higher in the early group—2100 (1350–2800) g—than in the late group—1790 (1270–2300) g—(*P* < 0.05) (Table 5).

**TABLE 5:**
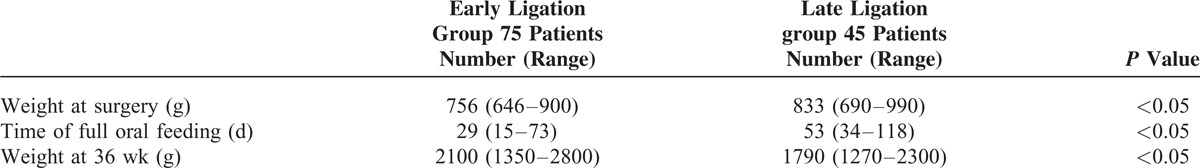
Nutritional Data

Regarding complications of surgery, among all surgical patients, 1 patient had postoperative left-sided pneumothorax necessitated chest tube insertion and resolved within few days, 1 patient developed recurrent laryngeal nerve paresis, and 12 patients died (5 in the early ligation group and 7 in the late group) at a median age of 29 (range 18–56) days of age due to complications not related to surgery (septicemia in 7, chronic lung disease in 3, interventricular hemorrhage grade IV in 1, and tension pneumothorax in 1). This number of postoperative complications and mortality were too small to be compared and analyzed statistically.

## DISCUSSION

There is no consensus on the optimal time of surgical intervention and no specific indications for elective surgical closure of PDA in preterm babies, predicting a favorable outcome in this high-risk group of patients.^8^

In our study, PDA ligation was performed successfully on 120 preterm infants who had medical failure or had contraindication to medical treatment. Short-term cardiorespiratory and nutritional outcomes were compared in early and late ligation groups, trying to find out the major differences that make one approach has more favorable outcome than the other.

Our study showed that when compared with surgical ligation performed at a later stage, patients with early surgical intervention required less oxygen postoperatively. This is explained simply as pulmonary overcirculation and subsequent alveolar edema might impair the pulmonary function if not relieved early and consequently increasing the oxygen need. This finding was observed before by Jaillard et al^9^ in their study in which they found less oxygen requirements in earlier PDA ligation patients.

Also, we found that infants in the early ligation group had earlier extubation since birth (fewer days of mechanical ventilation) but BPD incidence did not differ statistically. This is because early ligation of PDA improves pulmonary edema in premature infants,^10^ thus we can extubate the patients earlier, but BPD is a multifactorial disease. Yeh et al^11^ have suggested that the development of BPD is linked to the severity of respiratory distress syndrome and the high inspired oxygen concentrations delivered within 4 hours of age. For this reason, they proposed that any therapeutic regimen should be instituted very early to have any effect on reducing the development of BPD.

Farstad and Bratlid^12^ also found no difference in lung compliance and BPD with ductal closure in infants with respiratory distress syndrome. On the contrary, Szymankiewicz et al^10^ reported improved lung function after surgical closure of PDA. These data showed that the effect of closure of PDA on respiratory status in premature neonates is not clear regardless to the timing of surgery.

Nutritional status (weight, time of oral feeding restart) in our study was better with early PDA surgical closure (Table 5). This supports the concept that patency of the ductus arteriosus reduces intestinal blood flow while increasing the pulmonary blood flow (diastolic steal phenomenon).^13^ This concept was confirmed by Cassady et al^14^ who found that impaired intestinal blood flow may cause feeding intolerance and NEC in premature infants, and this can be significantly reduced by early prophylactic ligation of PDA in infants with extremely low birth weight. This can explain why we found early ligation of PDA led to a shorter duration of total parenteral nutrition sparing its serious complications. Jaillard et al^9^ have also shown that early surgical closure of the ductus arteriosus is associated with rapid achievement of full oral feeding and improved body growth, when compared with late surgical closure.

On contrary, a study by Jhaveri et al^5^ compared the outcomes of neonates who had PDA ligation by either an early approach or a conservative approach in 2 different periods and concluded that the conservative approach is associated with a lower rate of ductus ligation and earlier start of full oral feeding and less NEC. The authors did not discuss this finding and we cannot explain how neonates exposed to PDA for a longer duration are protected against NEC or how early surgical ligation can cause NEC.

Finally, we found that early surgical ligation is associated with improved short-term outcomes; hence, hospital stay and albit statistically non significant, lower mortality, this is in concordance with the findings of Jaillard et al.^9^

Indeed, some studies demonstrated that delaying surgical ligation might increase the likelihood of morbidity, mortality, or both.^15,16^

However, other studies reported that early surgical ligation of PDA in preterm infants can be associated with an increased risk for hospital morbidity and a worse long-term outcome.^17,18^ Because these data are observational, it is not known whether surgical ligation is really a contributor to morbidity and mortality, or it is just the severely compromised patients were referred for surgery.

The study had its limitations. Our study was not a randomized controlled trial but a retrospective observational one, so whatever results we got, it just shed light on the benefits of early approach of PDA closure rather than giving a strong recommendations for this strategy. Also we do not have long-term follow-up to see if these benefits were sustained.

## CONCLUSION

Early surgical ligation of symptomatic PDA who has failed medical treatment showed improvement in nutritional status (feeding tolerance and weight gain) and ventilatory status (early extubation and lower FiO2), with no influence on major outcomes.
