# Outcomes of Early Ligation of Patent Ductus Arteriosus in Preterms, Multicenter Experience: Erratum

**DOI:** 10.1097/01.md.0000469904.89299.d4

**Published:** 2015-07-17

In the article “Outcomes of Early Ligation of Patent Ductus Arteriosus in Preterms, Multicenter Experience”,^1^ which appeared in Volume 94, Issue 25 of *Medicine*, Dr. Enas A. A. Abdallah's name was inadvertently omitted, and Dr. Adnan A. Alsulaimani's name was incorrectly included. Dr. Mostafa A. Salama's name was also incorrectly listed as Mostafa Abdelazim. The article has since been corrected online.

## References

[1] Ibrahim MH Azab AA Kamal NM Outcomes of early ligation of patent ductus arteriosus in preterms, multicenter experience. *Medicine* 94 25:e915. 26107681 10.1097/MD.0000000000000915 PMC4504537

